# The two-hit hypothesis in acute esophageal necrosis: black esophagus in severe atherosclerosis and untreated gastroesophageal reflux disease: a case report

**DOI:** 10.1186/s13256-026-05899-y

**Published:** 2026-03-04

**Authors:** Sushrut Ingawale, Pujitha Vallivedu, Anchit Chauhan, Jack Jacob, Asim Haider, Prabin Sharma

**Affiliations:** 1 Department of Medicine, St. Vincent’s Medical Center, Quinnipiac University - Frank H. Netter MD School of Medicine, Bridgeport, CT 06606 USA; 2 Department of Medicine, Maulana Azad Medical College, New Delhi, DL India; 3 Department of Pathology, St. Vincent’s Medical Center, Quinnipiac University - Frank H. Netter MD School of Medicine, Bridgeport, CT USA; 4 Department of Gastroenterology, St. Vincent’s Medical Center, Quinnipiac University - Frank H. Netter MD School of Medicine, Bridgeport, CT USA

**Keywords:** Esophageal necrosis, Atherosclerosis, Esophagus, Necrosis, Reflux esophagitis, Case report, Risk factors

## Abstract

**Background:**

Acute esophageal necrosis, also known as “black esophagus,” is an uncommon but potentially life-threatening condition marked by diffuse circumferential necrosis of the esophageal mucosa. It typically presents with upper gastrointestinal bleeding, such as hematemesis or melena, in 85–90% of reported cases. Acute esophageal necrosis is often multifactorial in etiology and associated with underlying comorbidities including cardiovascular disease and esophageal mucosal injury. Owing to its rarity, atypical presentations of acute esophageal necrosis can delay diagnosis and appropriate management. This case is noteworthy for its nonclassical presentation and its support of the “two-hit” hypothesis for acute esophageal necrosis pathogenesis.

**Case presentation:**

A 71-year-old African American male with a medical history of coronary artery disease and untreated gastroesophageal reflux disease presented with odynophagia, epigastric discomfort, and nonradiating chest pain. He notably denied hematemesis or melena. Esophagogastroduodenoscopy revealed extensive circumferential black discoloration of the distal esophageal mucosa consistent with necrosis, along with duodenal ulcers. Biopsy findings confirmed the diagnosis of acute esophageal necrosis along with evidence of *Candida* infection. The patient was treated conservatively with intravenous proton pump inhibitors (esomeprazole), oral fluconazole, and sucralfate. A repeat esophagogastroduodenoscopy performed after several weeks demonstrated complete mucosal healing and resolution of necrosis.

**Conclusion:**

This case highlights an atypical, nonbleeding presentation of acute esophageal necrosis and reinforces the importance of maintaining clinical suspicion even in the absence of classic symptoms. It underscores the relevance of the “two-hit” hypothesis, where ischemic insult and impaired mucosal defense act synergistically to cause esophageal necrosis. Timely endoscopic evaluation and early initiation of appropriate therapy led to favorable outcomes in this high-risk patient. Increased awareness of such presentations can aid in earlier diagnosis and reduce the high morbidity and mortality associated with acute esophageal necrosis.

## Introduction

Acute esophageal necrosis (AEN), also known as black esophagus, Gurvits syndrome, esophageal stroke, or necrotizing esophagitis, is a rare but severe condition [1]. It is characterized by ischemic necrosis of the esophageal mucosa, which presents as a distinctly friable, and circumferential black discoloration. This necrosis can extend throughout to muscularis propria, and black discoloration typically extends to the gastroesophageal junction [2], where it abruptly transitions to normal tissue. AEN is most often associated with upper gastrointestinal bleeding, presenting as hematemesis, coffee-ground emesis, or melena [3]. The condition was first linked to upper gastrointestinal bleeding by Goldenberg et al. in 1990 and was further defined as a distinct syndrome by Gurvits et al. [1–3]. Diagnosis is confirmed through esophagogastroduodenoscopy (EGD), which reveals the characteristic necrotic mucosal changes. Despite its rarity, AEN is associated with a high mortality rate of approximately 32–38%, primarily in patients with multiple comorbidities and hemodynamic instability [4–6].

Although the syndrome typically presents with upper gastrointestinal bleeding in 85–90% of cases, atypical or nonbleeding presentations have been increasingly recognized in recent case reports. Prior descriptions of atypical AEN largely involve hemodynamic instability, diabetic ketoacidosis, or profound shock; however, reports of AEN presenting solely with odynophagia or chest discomfort without hematemesis, melena, or overt bleeding remain limited.

AEN results from multifactorial tissue insult, including hypoperfusion, gastric content reflux, and impaired local defenses. Key risk factors include older age (> 60 years), male gender, diabetes mellitus, cardiovascular diseases, sepsis, trauma, malnutrition, chronic kidney or liver diseases, and malignancy [2, 6]. The pathophysiology of AEN is unclear. However, with evolving literature with upcoming case reports citing varied associations, a “two-hit hypothesis” is emerging as the underlying mechanism instead of one single insult. AEN is now understood to arise from multifactorial tissue injury, and expanding literature supports a “two-hit” model involving an initial ischemic insult followed by impaired mucosal defenses, most often due to gastric acid exposure. This paradigm is particularly relevant in patients with significant atherosclerotic disease, chronic reflux, or other conditions predisposing to esophageal vulnerability.

The objective of this case report is to highlight an atypical, nonbleeding presentation of AEN in a high-risk patient and to illustrate how his clinical course supports the two-hit hypothesis. Additionally, this case is notable for demonstrating *Candida* superinfection in an immunocompetent host, an underreported but clinically important finding, and for documenting complete mucosal healing on follow-up endoscopy. By presenting this case, we aim to reinforce the need for clinical vigilance in patients without classic bleeding symptoms and provide insight into prognosis, potential superinfections, and sequelae such as stricture formation. Here, we present a case with severe atherosclerotic burden presenting with acute esophageal necrosis in presence of an untreated gastroesophageal reflux disease.

## Case capsule

A 71-year-old African American male presented with atypical chest pain, epigastric pain, odynophagia, nausea, vomiting, and loose stools but denied melena, hematemesis, fever, or dyspnea. His medical history included uncontrolled hypertension, diabetes, hyperlipidemia, obstructive coronary artery disease with stents, peripheral vascular disease requiring bilateral iliac artery stenting and a right femoral endarterectomy, hypertrophic obstructive cardiomyopathy, and untreated gastroesophageal reflux disease (GERD). He was an active smoker, a social drinker, and denied substance abuse.

**Fig. 1:**
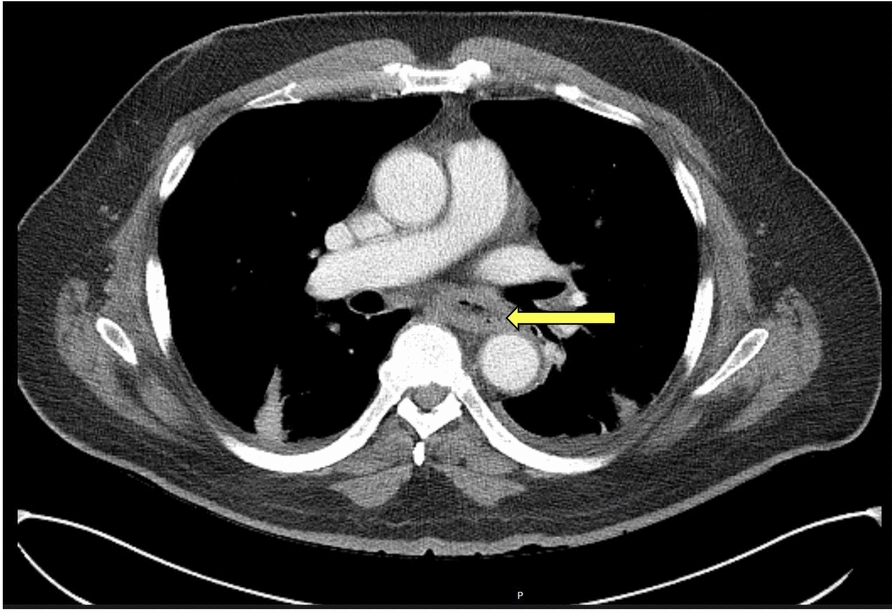
Computed tomography scan with intravenous contrast revealing a thick-walled esophagus (arrow) suggestive of esophagitis along with debris and fluid within the lumen, without any evidence of perforation

**Fig. 2:**
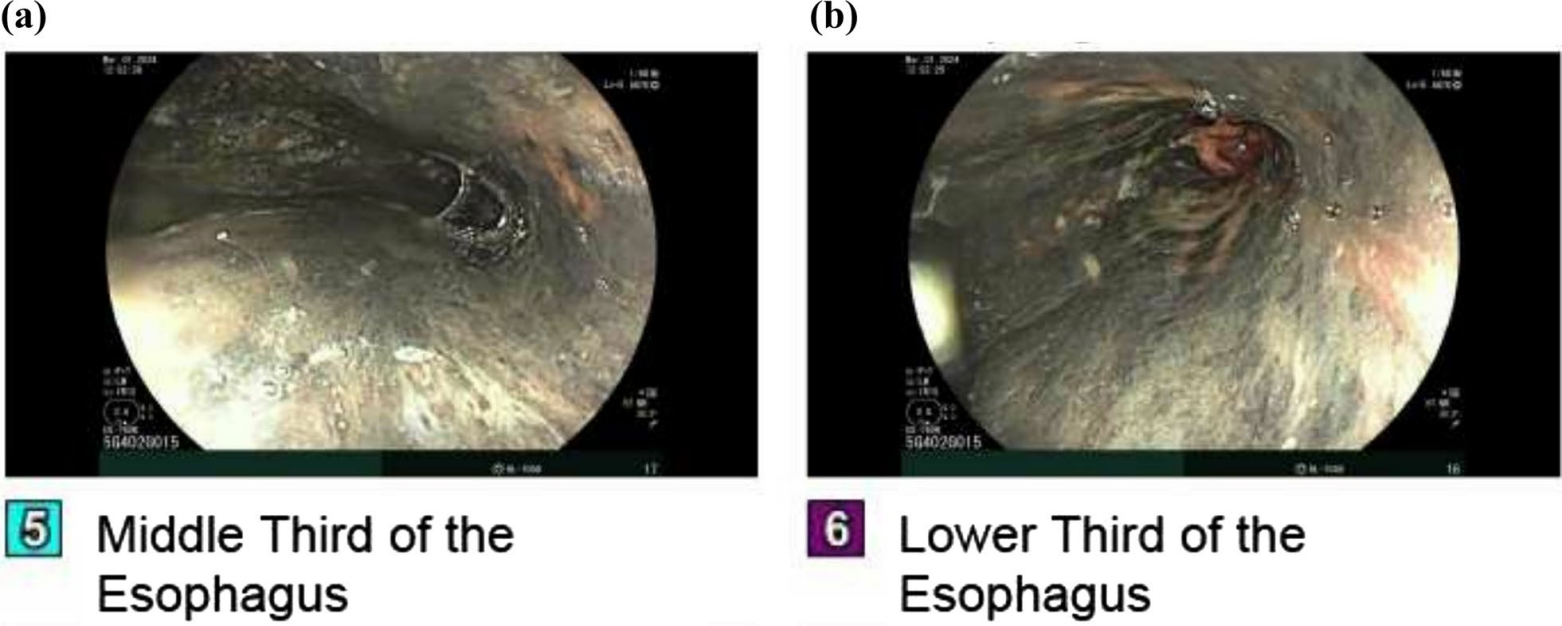
Esophagogastroduodenoscopy at the time of presentation, revealing diffuse mucosal changes characterized by blackish discoloration of the middle (**a**) and lower third (**b**) of the esophagus extending from 24 to 40 cm from the incisors

**Fig. 3:**
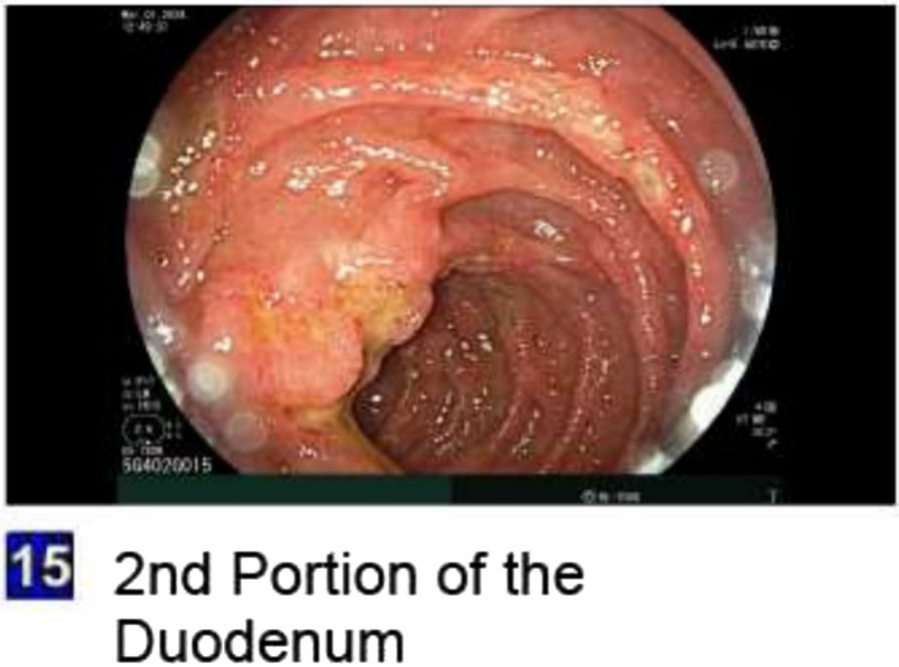
Esophagogastroduodenoscopy at the time of presentation revealing multiple nonbleeding superficial duodenal ulcers with no stigmata of bleeding in the duodenum

**Fig. 4:**
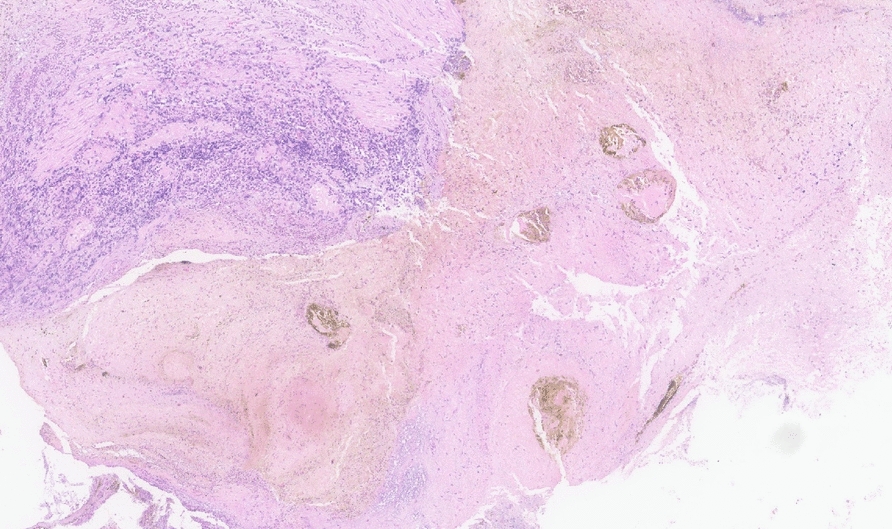
Low power (10× magnification) view showing ulcerated and necrotic esophageal mucosa with inorganic debris and a mixed inflammatory cell infiltrate

**Fig. 5:**
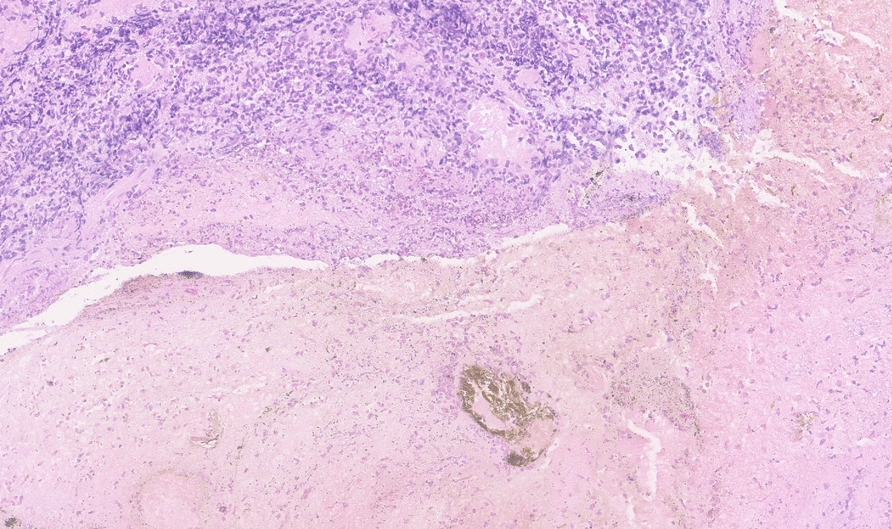
High power (40× magnification) view showing the gangrenous basaloid epithelium and necrotic debris

On physical examination, the patient was tachycardic and had mild abdominal tenderness in the epigastric region, without signs of peritonitis. Laboratory findings demonstrated leukocytosis (17,300 cells/µL), elevated hemoglobin (18.6 g/dL), and newly elevated serum creatinine (3.0 mg/dL) and blood urea nitrogen (51 mg/dL). The urine toxicology screen was negative. Electrocardiogram revealed no acute cardiac ischemic changes. Troponin was mildly elevated at 97 ng/L, prompting cardiac monitoring with telemetry which showed no arrhythmias or ischemic changes. Serial troponins remained stable, ruling out acute coronary syndrome. Intravenous fluids were administered to correct dehydration with normalization of renal function. A computed tomography (CT) scan of the chest with intravenous contrast revealed features suggestive of esophagitis (Fig. 1). The esophagogastroduodenoscopy revealed extensive circumferential mucosal black discoloration in the middle and lower third of the esophagus and multiple nonbleeding superficial ulcers in the duodenum (Figs. 2, 3). The biopsy showed ulcerated and necrotic esophageal mucosa with inorganic debris and a mixed inflammatory cell infiltrate confirming the diagnosis of AEN (Figs. 4, 5). A Periodic acid–Schiff stain smear was positive for scanty *Candida* yeasts.

**Fig. 6:**
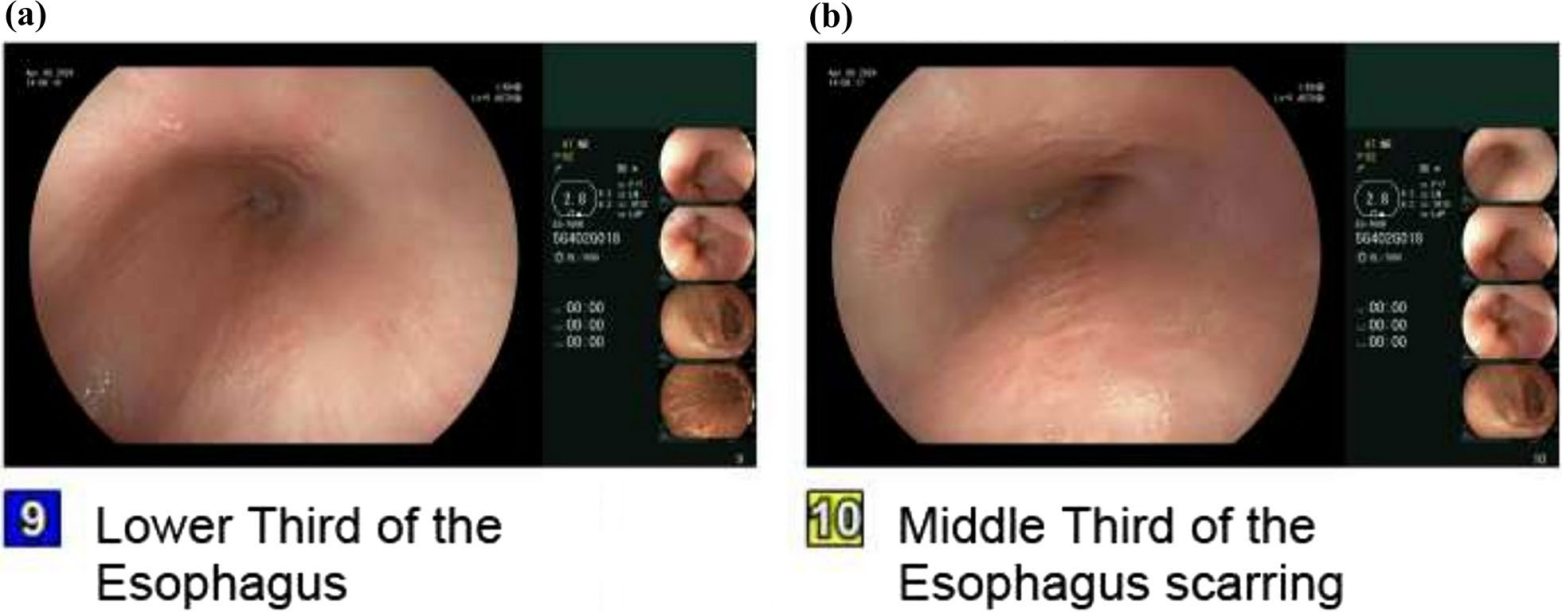
Interval follow-up esophagogastroduodenoscopy after 6 weeks from presentation, revealing healed esophageal mucosa with some residual scarring of the middle (**a**) and lower third (**b**) of the esophagus

In the absence of perforation, the patient was managed medically with intravenous pantoprazole 40 mg twice daily, later transitioned to oral esomeprazole 40 mg twice daily with sucralfate suspension four times daily. A 14-day course of oral fluconazole 200 mg daily was administered. The patient was advised to follow a bland, soft diet to reduce esophageal irritation. Follow-up EGD at 6 weeks showed complete healing of ulcers and pathologies with some residual scarring (Fig. 6).

## Discussion

Acute esophageal necrosis (AEN) has a prevalence of 0.001–0.28% and predominantly affects older adults, typically in their sixties (average age 67 years), with a male-to-female ratio of 4:1 [1, 7, 8]. It involves the distal esophagus in 97% of cases, likely owing to its relatively poor vascular supply [9]. AEN is often accompanied by duodenal bulb ulcers, as both structures share blood supply from the celiac trunk [10, 11]. Contributing factors include low esophageal sphincter pressure, gastric reflux, ischemia, and impaired mucosal repair [2, 6]. On the basis of an extensive search review, the Table 1 summarizes common associations and triggers as proposed “first” and “second” hits in the pathophysiology of AEN. Notably, AEN has been linked to diabetes mellitus (24%), malignancy (20%), hypertension (20%), and alcohol abuse (10%). AEN should be considered in patients presenting with upper gastrointestinal bleeding, particularly those with risk factors such as diabetes, shock, or sepsis. Other conditions that can present with black mucosal discoloration, including malignant melanoma, caustic burns, dye ingestion, pseudomelanosis, and coal dust exposure, should be ruled out [2].

**Table 1:** Etiology of acute esophageal necrosis [2, 5, 6, 12–14, 17–19]

Category	Types	Examples
Underlying conditions (first hit)	Systemic conditions	Older age (60+ years), males, diabetes mellitus, hypertension, dyslipidemia, malnutrition, alcohol abuse, malignancy, immunosuppression, trauma
	Cardiovascular diseases	Coronary heart disease, peripheral artery disease, congestive heart failure
	Pulmonary and renal diseases	Chronic obstructive pulmonary disease, chronic kidney disease
	Hepatic disorders	Liver cirrhosis, alcoholic hepatitis
	Hemodynamic and vascular events	Hemodynamic compromise, hypovolemia, sepsis, acute myocardial infarction, cardiac arrest, stress cardiomyopathy, acute blood loss, aortic dissection, thromboembolism
Precipitating Factors (second hit)/associated factors	Gastrointestinal conditions	Gastric reflux as in gastroesophageal reflux disease
	Metabolic causes	Diabetic ketoacidosis, lactic acidosis, adrenal insufficiency
	Infections	Pneumonia, ischemic colitis, spontaneous bacterial peritonitis, secondary peritonitis, necrotizing fasciitis, osteomyelitis, endocarditis, infectious esophagitis (HSV 1, CMV, *Candida*, and so on), AKI, sepsis, shock, *Candida*
	Procedural and iatrogenic	Surgery, chemotherapy, solid organ transplantation, gastric volvulus, achalasia, drugs (NSAIDs, bisphosphonates, and so on), percutaneous procedures (for example, gastrostomy, coronary intervention), sodium polystyrene sulfonate (kayexalate) use, endoscopic retrograde cholangiopancreatography
	Other causes	Stevens–Johnson syndrome, Henoch–Schönlein purpura, antiphospholipid syndrome, acute fatty liver of pregnancy, hepatic encephalopathy, Wernicke’s encephalopathy, pneumomediastinum, erythema multiforme, anticardiolipin antibodies, hypersensitivity

HSV 1, herpes simplex virus; CMV, cytomegalovirus; AKI, acute kidney injury; NSAID, nonsteroidal anti inflammatory drugs

Our case supports the “two-hit” hypothesis: an initial vascular insult (for example, low-flow state) and a subsequent insult from acid reflux [6]. The patient had severe atherosclerosis (first hit), including obstructive coronary artery disease with stents, peripheral vascular disease requiring bilateral iliac artery stenting, and femoral endarterectomy, along with uncontrolled hypertension and active smoking. His atherosclerotic cardiovascular disease (ASCVD) risk was calculated at 63.6% for a cardiovascular event (coronary or stroke death or nonfatal myocardial infarction or stroke) in the next 10 years and 8.9% risk of 10-year cardiovascular risk if risk factors were optimal. Additionally, untreated GERD and poor dietary habits, including lying down after meals, may have predisposed him to chronic esophageal irritation (second hit).

This case was atypical for AEN, as the patient lacked hematemesis or melena, seen in 85–90% of cases [3, 12]. Instead, he presented with odynophagia, atypical chest pain, and epigastric discomfort. He also lacked diabetic ketoacidosis, a common association [12–14], but had acute kidney injury, reported in only 20% of cases [3]. These findings highlight the variability of AEN and the need for high clinical suspicion even in the absence of classic symptoms.

Although *Candida* species are well-recognized opportunistic pathogens in immunocompromised individuals, superinfection in the setting of AEN is less frequently reported. Existing literature suggests that fungal colonization may occur in up to 5–10% of AEN cases but is often underdiagnosed because biopsies are not routinely obtained. *Candida* may exacerbate mucosal injury through the direct invasion of already ischemic tissue and may increase the risk of secondary infection, delayed healing, or rare complications such as sepsis. In immunocompetent patients, as in this case, the presence of *Candida* likely reflects severe mucosal barrier disruption rather than underlying immunosuppression. Nevertheless, the identification of fungal elements warrants targeted antifungal therapy, as timely treatment may prevent further luminal injury and systemic dissemination.

Duodenal involvement supports a vascular etiology, given the shared celiac trunk blood supply [10, 11]. Based on Gurvits *et al*.’s 2010 AEN staging (Table 2), this case corresponds to stage 1. The presence of *Candida* in the esophageal biopsy, though uncommon, is clinically significant as it may lead to severe complications like sepsis [3, 5, 15]. Follow-up endoscopy confirmed healing, underscoring the importance of reassessing for sequelae such as perforation, stricture formation (> 10%), or persistent ulceration [16]. Given AEN’s rapid progression and high mortality, early diagnosis and intervention are crucial for improving outcomes. Follow-up endoscopy in this patient demonstrated complete mucosal healing, which is an important positive prognostic marker. Most patients with AEN who survive the initial insult show significant mucosal recovery within weeks; however, esophageal stricture formation is reported in more than 10% of cases. Strictures typically present within 3–8 weeks after the acute event and may require endoscopic dilation. Documenting post-recovery endoscopic findings, therefore, not only confirms healing but also ensures early detection of late complications. The absence of stricture in this case further supports a favorable clinical trajectory, likely attributable to prompt acid suppression, bowel rest, and management of precipitating factors.

**Table 2:** Endoscopic staging of acute esophageal necrosis (AEN) by Gurvits *et al*. [12]

Stage	Endoscopic appearance	Time frame
Stage 0	Normal mucosa and esophagus; intact epithelium	–
Stage 1	Blackened, circumferential mucosa; yellow exudates possible	At the time of reporting
Stage 2	Pink mucosa with residual dark spots; thick, white exudates that strip off easily	1–4 weeks after diagnosis
Stage 3	Normal pink mucosa or areas of granulation tissue	1–2 weeks after diagnosis

## Conclusion

This case underscores that AEN can occur even in the absence of gastrointestinal bleeding and further reinforces the two-hit hypothesis involving ischemia and mucosal injury. Broader clinical awareness of such atypical presentations is essential, as early endoscopic evaluation and targeted therapy can significantly influence outcomes in this potentially life-threatening condition.

## Data Availability

Available upon request to authors.

## Abbreviations

AEN: Acute esophageal necrosis
EGD: Esophagogastroduodenoscopy
GERD: Gastroesophageal reflux disease
MI: Myocardial Infarction
ASCVD: Atherosclerotic cardiovascular disease

## References

[1] Gurvits GE, Shapsis A, Lau N, Gualtieri N, Robilotti JG. Acute esophageal necrosis: a rare syndrome. J Gastroenterol. 2007;42(1):29–38. 10.1007/s00535-006-1974-z. 17322991 10.1007/s00535-006-1974-z

[2] Rehman O, Jaferi U, Padda I, Khehra N, Atwal H, Parmar M. Epidemiology, pathogenesis, and clinical manifestations of acute esophageal necrosis in adults. Cureus. 2021;13(7): e16618. 10.7759/cureus.16618. 34447648 10.7759/cureus.16618 PMC8381445

[3] Schizas D, Theochari NA, Mylonas KS, *et al*. Acute esophageal necrosis: a systematic review and pooled analysis. World J Gastrointest Surg. 2020;12(3):104–15. 10.4240/wjgs.v12.i3.104. 32218893 10.4240/wjgs.v12.i3.104 PMC7061242

[4] Day A, Sayegh M. Acute oesophageal necrosis: a case report and review of the literature. Int J Surg. 2010;8(1):6–14. 10.1016/j.ijsu.2009.09.014. 19800431 10.1016/j.ijsu.2009.09.014

[5] Dias E, Santos-Antunes J, Macedo G. Diagnosis and management of acute esophageal necrosis. Ann Gastroenterol. 2019;32(6):529–40. 10.20524/aog.2019.0418. 31700229 10.20524/aog.2019.0418 PMC6826069

[6] Rycyk-Bojarzyńska A, Kasztelan-Szczerbińska B, Cichoż-Lach H. Into the dark of the black oesophagus. Ann Agric Environ Med. 2024;31(3):450–4. 10.26444/aaem/176295. 39344738 10.26444/aaem/176295

[7] Augusto F, Fernandes V, Cremers MI, *et al*. Acute necrotizing esophagitis: a large retrospective case series. Endoscopy. 2004;36(5):411–5. 10.1055/s-2004-814318. 15100949 10.1055/s-2004-814318

[8] Grudell ABM, Mueller PS, Viggiano TR. Black esophagus: report of six cases and review of the literature, 1963–2003. Dis Esophagus. 2006;19(2):105–10. 10.1111/j.1442-2050.2006.00549.x. 16643179 10.1111/j.1442-2050.2006.00549.x

[9] Siddiqi A, Chaudhary FS, Naqvi HA, Saleh N, Farooqi R, Yousaf MN. Black esophagus: a syndrome of acute esophageal necrosis associated with active alcohol drinking. BMJ Open Gastroenterol. 2020;7(1): e000466. 10.1136/bmjgast-2020-000466. 32788199 10.1136/bmjgast-2020-000466 PMC7422689

[10] V S, A D, S L, L B. Black esophagus: acute esophageal necrosis due to alcohol intoxication. Trop Gastroenterol. 2017;38(1):47–8. 10.7869/tg.391.

[11] Martins D, Marques R, Costa P, de Sousa JP. The dark side of the esophagus. Autops Case Rep. 2021;11: e2021284. 10.4322/acr.2021.284. 34307235 10.4322/acr.2021.284 PMC8214898

[12] Gurvits GE. Black esophagus: acute esophageal necrosis syndrome. World J Gastroenterol. 2010;16(26):3219–25. 10.3748/wjg.v16.i26.3219. 20614476 10.3748/wjg.v16.i26.3219 PMC2900712

[13] Pollis RM, Furlanetto D, Pagin E, Scaroni C, Barbot M, Voltan G. Ketosis-prone diabetes presenting with acute esophageal necrosis or “black esophagus”: an intriguing new clinical association. Endocr Metab Immune Disord Drug Targets. 2024;24(7):857–63. 10.2174/0118715303279019231127065331. 38083890 10.2174/0118715303279019231127065331

[14] Thuluvath AJ, Moore D, Alghanim F. Acute esophageal necrosis: a cause or effect of diabetic ketoacidosis (DKA)?: 1649. Official journal of the American College of Gastroenterology | ACG. 2017;112:S891.

[15] Sharma V, De A, Ahuja A, Lamoria S, Lamba BMS. Acute esophageal necrosis caused by candidiasis in a patient with systemic lupus erythematosus. J Emerg Med. 2016;51(1):77–9. 10.1016/j.jemermed.2016.04.005. 27221020 10.1016/j.jemermed.2016.04.005

[16] Abdullah HM, Ullah W, Abdallah M, Khan U, Hurairah A, Atiq M. Clinical presentations, management, and outcomes of acute esophageal necrosis: a systemic review. Expert Rev Gastroenterol Hepatol. 2019;13(5):507–14. 10.1080/17474124.2019.1601555. 30933549 10.1080/17474124.2019.1601555

[17] Kitagawa K, Masuda H, Mitoro A, *et al*. Black esophagus: a life-threatening adverse event associated with endoscopic retrograde cholangiopancreatography. Clin Endosc. 2024;57(2):270–3. 10.5946/ce.2023.047. 37524562 10.5946/ce.2023.047 PMC10984737

[18] Kram M, Gorenstein L, Eisen D, Cohen D. Acute esophageal necrosis associated with gastric volvulus. Gastrointest Endosc. 2000;51(5):610–2. 10.1016/S0016-5107(00)70304-X. 10805856 10.1016/s0016-5107(00)70304-x

[19] Garcia Rodriguez V, Grami Z, Laney J, Cai Z, Larson S. Esophageal necrosis associated with sodium polystyrene sulfonate (Kayexalate) use. Proc (Bayl Univ Med Cent). 2020;33(4):624–6. 10.1080/08998280.2020.1801322. 33100548 10.1080/08998280.2020.1801322 PMC7549977

